# Crystal Ball in a Blood’s Drop: Unlocking Hidden Prognostic Power in the Neutrophil-to-Lymphocyte Ratio (NLR) and the Platelet-to-Lymphocyte Ratio (PLR) for Elderly Hip Fracture Patients

**DOI:** 10.3390/jcm14103584

**Published:** 2025-05-20

**Authors:** Andrea Perna, Giuseppe Rovere, Marco Passiatore, Andrea Franchini, Luca Macchiarola, Francesco Maruccia, Raffaele Vitiello, Franco Lucio Gorgoglione

**Affiliations:** 1 Department of Orthopedics and Trauma Surgery, Fondazione Casa Sollievo Della Sofferenza IRCCS, 71013 San Giovanni Rotondo, Italy; a.franchini@operapadrepio.it (A.F.); luca.macchiarola@hotmail.it (L.M.); francescomaruccia@googlemail.com (F.M.);; 2 Clinical Science and Translational Medicine, Section of Orthopaedics and Traumatology, University of Rome Tor Vergata, 00133 Rome, Italy; giuseppe.rovere02@icatt.it; 3 Bone and Joint Surgery Department, University of Brescia, ASST-Spedali Civili, 25123 Brescia, Italy; passiatore.m@gmail.com; 4 Department of Orthopedics and Geriatric Sciences, Catholic University of the Sacred Heart, 00168 Rome, Italy; lele.vitiello@gmail.com

**Keywords:** hip fractures, neutrophil-to-lymphocyte ratio, platelet-to-lymphocyte ratio, PLR, NLR, elderly, mortality

## Abstract

**Background/Objectives:** Hip fractures in elderly patients are associated with high morbidity and mortality, requiring early risk stratification to optimize management. Systemic inflammatory markers such as the neutrophil-to-lymphocyte ratio (NLR) and the platelet-to-lymphocyte ratio (PLR) have emerged as potential prognostic tools. This study aimed to evaluate the predictive value of the NLR and PLR measured at admission for adverse outcomes following hip fracture surgery in elderly patients. **Methods:** This retrospective, single-center cohort study included patients aged 65 years or older admitted for hip fractures between January 2019 and December 2023. Baseline demographic, clinical, surgical, and laboratory data were collected. Primary outcomes were 30-day, 90-day, and 1-year mortality; secondary outcomes included postoperative ICU admission and prolonged hospitalization (>15 days). Univariable and multivariable Cox regression analyses were performed. Receiver operating characteristic (ROC) curve analysis determined optimal cut-offs for the NLR and PLR. **Results:** Among 395 included patients (mean age 84 years, 56.4% female), the 30-day, 90-day, and 1-year mortality rates were 4.8%, 10.5%, and 13.9%, respectively. ROC analysis identified cut-offs of 7.2 for the NLR (AUC 0.78, sensitivity 69.7%, specificity 85.4%) and 189.4 for the PLR (AUC 0.73, sensitivity 65.1%, specificity 76.1%). Elevated NLR and PLR were independently associated with increased risk of mortality, ICU admission, and prolonged hospitalization. **Conclusions:** Elevated NLR and PLR at admission are independent, strong predictors of adverse outcomes in elderly patients with hip fractures. These inexpensive, readily available biomarkers could enhance early risk stratification and inform perioperative management strategies.

## 1. Introduction

Hip fractures among the elderly constitute a major global health challenge, resulting in considerable morbidity, functional decline, extended hospital stays, and high mortality rates [1,2]. As populations age, the incidence of hip fractures rises globally, posing significant implications for patients and healthcare systems [3,4]. Over 300,000 Americans aged 65 and older sustain hip fractures annually, with similar trends elsewhere [3,4]. Age-related frailty makes this group highly vulnerable to postoperative complications, underscoring the need for early risk stratification upon hospital admission [5,6]. Conventional predictors such as advanced age, comorbidities (Charlson Comorbidity Index, CCI), nutritional deficiencies (hypoalbuminemia), and cardiovascular biomarkers (N-terminal pro–B-type natriuretic peptide NT-proBNP) are established indicators of poor outcomes following surgery [5,6,7]. However, the role of systemic inflammatory responses in recovery needs further exploration [8]. Growing evidence emphasizes inflammatory markers as prognostic tools across surgical fields [9,10,11]. Among these, the neutrophil-to-lymphocyte ratio (NLR) and platelet-to-lymphocyte ratio (PLR) have gained attention. An elevated NLR reflects a heightened pro-inflammatory state with neutrophilia and lymphopenia, linked to infection, poor healing, and worse prognosis [9,10,12]. Similarly, high PLR indicates systemic inflammation and altered thrombosis, contributing to perioperative complications [12,13]. Despite research into the NLR and PLR in cancer, cardiovascular, and infectious diseases [9,10,11,12,13], their prognostic role in hip fracture surgery remains underexplored [14]. This burden is expected to grow substantially in the coming decades due to demographic shifts. With the global population aging rapidly, the incidence of fragility fractures, particularly femoral fractures, is projected to increase significantly [2,3]. This trend is especially concerning for health systems already under pressure from resource constraints, making effective triage and risk assessment strategies more vital than ever [15]. A refined evaluation of the NLR and PLR could offer cost-effective tools for early risk identification and personalized perioperative care. Importantly, NLR and PLR are derived from simple, inexpensive, and widely available blood tests routinely performed upon hospital admission. Unlike advanced imaging or specialized biomarkers, these ratios can be calculated without additional costs or time delays, providing clinicians with immediate insights into the patient’s inflammatory and immune status [16]. This economic accessibility makes them particularly suitable for integration into standard preoperative workflows, especially in resource-limited or high-volume settings. Having reliable, rapid, and low-cost biomarkers to identify patients at increased risk of postoperative complications could fundamentally improve clinical decision-making [16]. Stratifying risk early would allow healthcare providers to selectively allocate intensive monitoring, rehabilitation resources, and specialized care pathways to those most likely to benefit. In the context of rising case numbers, this targeted strategy not only enhances individual outcomes but also improves the efficiency and sustainability of healthcare delivery.

Addressing this knowledge gap is crucial given the clinical and economic burden of hip fractures. A refined evaluation of the NLR and PLR could offer cost-effective tools for early risk identification and personalized perioperative care. Recognizing high-risk patients via these accessible biomarkers may enhance monitoring, pre-emptive intervention, and resource allocation, improving outcomes and reducing healthcare strain. This study aims to investigate the associations between the NLR and PLR at emergency admission and short-term (30-day, 90-day) and long-term (1-year, overall) mortality, ICU admissions, and prolonged hospital stays (over 15 days) in elderly patients undergoing hip fracture surgery. By clarifying their predictive value, we hope to provide clinicians with practical, evidence-based tools for optimizing patient management.

## 2. Materials and Methods

### 2.1. Study Design and Patient Selection

This retrospective, single-center cohort study included patients admitted to the emergency department of our institution with hip fractures between 1 January 2019 and 31 December 2023. This study adhered to the STROBE (Strengthening the Reporting of Observational Studies in Epidemiology) guidelines [17]. The study protocol was conducted in accordance with the ethical principles of the Declaration of Helsinki (1964) and its subsequent amendments. Following extensive consultation with the internal departmental Institutional Review Board (IRB) and in consideration of the retrospective, non-interventional nature of this study, as well as the fact that all patients received treatment in accordance with the standard of care defined by our institution, formal ethical committee approval was deemed unnecessary. Informed consent for the scientific use of anonymized clinical data was obtained in compliance with institutional regulations and the guidelines of the Italian Data Protection Authority. All patient data were fully anonymized to safeguard confidentiality. The authors used ChatGPT (OpenAI, GPT-4) for the purposes of language editing.

### 2.2. Inclusion and Exclusion Criteria

Eligible participants were aged 65 years or older and sustained a hip fracture following a low-energy trauma, defined as a fall from standing height or lower. Patients were excluded if they were deemed inoperable, had pathological fractures, presented with established sepsis at admission, had previous surgery for femoral neck fracture, or were affected by advanced malignancies.

### 2.3. Data Collection and Database Formations

At admission, all patients were registered in a standardized institutional database capturing demographic information (sex, age), clinical variables (CCI, type of fracture), and pre-existing anticoagulant therapy. For this analysis, additional surgical data (type of surgery performed, time from emergency admission to surgery) and laboratory parameters measured at admission were extracted. Laboratory data included complete blood count (used to calculate NLR and PLR according Vitiello et al. [8]), hemoglobin concentration, serum albumin level (hypoalbuminemia defined as albumin <3.5 g/dL), and NT-proBNP values (a cut-off value of 1099 ng/L was established according to Zhang et al. [7]). Time to surgery was treated as both a continuous and categorical variable, distinguishing early (<48 h) versus delayed (≥48 h) interventions. Length of stay (LoS) was recorded as a continuous measure, with a prolonged hospitalization defined as a stay exceeding 15 days.

### 2.4. Outcome Measures

Primary endpoints were 30-day, 90-day, and 1-year all-cause mortality. Secondary outcomes included the necessity of postoperative admission to the intensive care unit (ICU) and prolonged hospitalization (LoS > 15 days). Mortality data were collected through hospital records and follow-up contact with primary care physicians or relatives when necessary.

### 2.5. Statistical Analysis

All statistical analyses were performed using SPSS Statistics software, version 26.0 (IBM Corp., Armonk, NY, USA). Data completeness was assessed, and missing continuous values (<3% of cases) were imputed using multiple imputation by chained equations, ensuring robust variance estimates. Continuous variables were assessed for normality using the Kolmogorov–Smirnov test. Normally distributed data were expressed as mean and standard deviation (SD) and compared using the independent samples *t*-test. Non-normally distributed variables were presented as median and interquartile range (IQR) and analyzed using the Mann–Whitney U test. Categorical variables were reported as frequencies and percentages and compared using the chi-square test or Fisher’s exact test where appropriate. Univariable Cox proportional hazards regression analyses were conducted to explore associations between candidate predictors and outcomes (30-day and 90-day mortality, ICU need, and prolonged LoS). Predictors with a *p*-value < 0.10 in univariable analyses were selected for multivariable Cox regression models to identify independent risk factors for 1-year and total mortality. Model assumptions, including proportional hazards, were tested using Schoenfeld residuals. The predictive performances of inflammatory markers (NLR, PLR) for 30-day mortality and ICU need were evaluated using receiver operating characteristic (ROC) curve analyses, and optimal thresholds were determined using the Youden Index. NLR and PLR cut-off values were established at 7.2 and 189.4, respectively. Hazard ratios (HRs) with 95% confidence intervals (CIs) were calculated. A two-sided *p*-value of <0.05 was considered statistically significant for all tests.

## 3. Results

### 3.1. Baseline Characteristics

The baseline demographic and clinical characteristics of the study population are summarized in Table 1. Among the records reviewed for 632 patients, only 395 individuals met the predefined inclusion and exclusion criteria and were consequently included in the final analysis. The cohort had a mean age of 84 years (SD 12.3), and females represented 56.4% (*n* = 223) of the sample, consistent with the known higher incidence of osteoporotic fractures in older women. Comorbidity burden, assessed using the CCI, was distributed as follows: 36.8% (*n* = 144) had a CCI of 0, indicating no recorded comorbid conditions; 34.2% (*n* = 137) had a CCI of 1; and 29.0% (*n* = 114) had a CCI greater than 2, reflecting a substantial presence of multimorbidity in nearly one-third of the population. Regarding fracture classification, intertrochanteric fractures were the most common (46.7%, *n* = 185), followed by subcapital fractures (28.5%, *n* = 112) and basi-cervical fractures (24.8%, *n* = 98). Surgical management primarily involved internal fixation, performed in 64.3% (*n* = 253) of cases, while hemiarthroplasty and total hip arthroplasty accounted for 18.3% (*n* = 73) and 13.6% (*n* = 54), respectively. Cannulated screw fixation was utilized in a small subset of patients (3.8%, *n* = 15). The median time from admission to surgery was 2.9 days (interquartile range, IQR: 0.5–5.9). A total of 289 patients (72.4%) underwent surgery within 48 h, aligning with current clinical guidelines favoring early surgical intervention, while 106 patients (27.6%) were operated on after 48 h. Pre-trauma anticoagulant therapy was documented in 24.2% (*n* = 93) of patients, an important consideration for perioperative management. Inflammatory and biochemical parameters revealed a median NLR of 6.73 (IQR: 2.4–9.2) and a median PLR of 122.5 (IQR: 112.5–197.8). Median hemoglobin was 10.2 g/dL (IQR: 6.7–14.9), indicating a tendency toward preoperative anemia, commonly observed in geriatric trauma populations. NT-proBNP levels were markedly variable, with a median of 390.5 ng/L (IQR: 187.3–14,573.2), underscoring the heterogeneity in cardiac function. Median serum albumin was 4.6 g/dL (IQR: 2.5–5.2), and hypoalbuminemia, defined as albumin <3.5 g/dL, was present in 21.3% (*n* = 82) of patients. Mortality outcomes showed that 30-day mortality occurred in 4.8% (*n* = 19) of the cohort, 90-day mortality in 10.5% (*n* = 40), and 1-year mortality in 13.9% (*n* = 53). The total mortality rate during the entire follow-up period was 28% (*n* = 112). Regarding postoperative complications, ICU admission was necessary in 9.6% (*n* = 37) of cases. Prolonged hospitalization, defined as a stay longer than 15 days, was observed in 15.9% (*n* = 61) of cases. The mean hospital length of stay was 9 days (SD 3.2), which reflects both the medical complexity and rehabilitation needs associated with geriatric hip fractures.

### 3.2. NLR and PLR Cut-Off Value Determination

**Figure 1:**
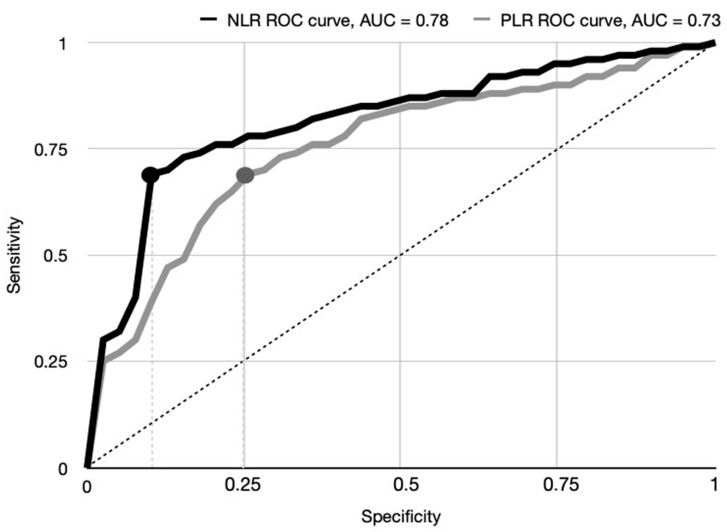
Receiver operating characteristic (ROC) curves illustrating the predictive performance of the neutrophil-to-lymphocyte ratio (NLR) and the platelet-to-lymphocyte ratio (PLR) for 30-day mortality. The area under the ROC curve (AUC) for the NLR was 0.78 [95% CI 0.66–0.81], indicating good discriminatory power. The AUC for the PLR was 0.73 [95% CI 0.61–0.79], suggesting moderate discriminatory ability. The dashed line represents the line of no discrimination (AUC = 0.5). The dot on the NLR ROC curve represents the cut-off value of 7.2. The dot on the PRL ROC curve represents the cut-off value of 189.4.

Univariate analysis followed by ROC curve analysis was performed to identify optimal cut-off values for the NLR and PLR in predicting the primary outcome of 30-day mortality. This analysis yielded a cut-off value of 7.2 for the NLR and 189.4 for the PLR. The area under the ROC curve (AUC) for the NLR was 0.78 [95% CI 0.66–0.81], indicating good discriminatory power for 30-day mortality. Specifically, the sensitivity prediction of the NLR > 7.2 was 69.7%, and the specificity was 85.4%. The AUC for the PLR was 0.73 [95% CI 0.61–0.79], suggesting moderate discriminatory ability. A PLR > 189.4 demonstrated a sensitivity of 65.1% and a specificity of 76.1%. These ROC curve findings concerning primary outcome are visually represented in Figure 1.

### 3.3. Univariate Analysis: Initial Assessment of Predictors

The univariable Cox regression analysis, summarized in Table 2, confirmed the significant association between several classical clinical predictors and adverse outcomes. Advanced age, a CCI greater than 2, delayed surgical intervention, hypoalbuminemia, and elevated NT-proBNP levels were all associated with increased mortality, a higher likelihood of ICU admission, and a longer hospital stay. Importantly, particular emphasis was placed on the prognostic impact of inflammatory biomarkers. Patients with an NLR greater than 7.2 had a hazard ratio (HR) of 2.38 (95% CI 2.01–2.75; *p* = 0.001) for 30-day mortality, corresponding to a 138% increase in risk compared to those with lower NLR values. The risk of 90-day mortality was similarly elevated by 92% (HR 1.92; 95% CI 1.23–2.13; *p* = 0.003) among patients with an elevated NLR. In terms of postoperative outcomes, an NLR above the established threshold was associated with a 152% increased likelihood of requiring ICU admission (HR 2.52; 95% CI 2.21–2.87; *p* = 0.0023) and a 97% increase in the risk of prolonged hospitalization (HR 1.97; 95% CI 1.23–2.17; *p* = 0.002). The PLR demonstrated a similar predictive trend. Patients with a PLR greater than 189.4 had a 99% higher risk of 30-day mortality (HR 1.99; 95% CI 1.73–2.31; *p* = 0.002) and a 78% higher risk of 90-day mortality (HR 1.78; 95% CI 1.43–1.99; *p* = 0.002). An elevated PLR was also associated with a 65% increased likelihood of ICU admission (HR 1.65; 95% CI 1.23–1.89; *p* = 0.0021) and a 54% increased risk of a hospital stay exceeding 15 days (HR 1.54; 95% CI 1.21–1.78; *p* = 0.0052).

### 3.4. Multivariable Analysis: NLR and PLR as Independent Predictors

Multivariable Cox regression analyses, presented in Table 3, were conducted to adjust for confounders including age, comorbidity burden, timing of surgery, hypoalbuminemia, and NT-proBNP levels. In these models, both NLR and PLR retained their significance as independent predictors of mortality. An NLR greater than 7.2 remained associated with a 98% increased risk of 1-year mortality (HR 1.98; 95% CI 1.73–2.15; *p* = 0.001) and a 95% higher risk of overall mortality across the study period (HR 1.95; 95% CI 1.27–2.23; *p* = 0.003). Similarly, a PLR above 189.4 independently predicted an 81% increase in 1-year mortality risk (HR 1.81; 95% CI 1.61–2.23; *p* = 0.032) and a 68% increase in total mortality risk (HR 1.68; 95% CI 1.29–1.83; *p* = 0.029). As expected, traditional clinical predictors also demonstrated robust independent associations. A CCI greater than 2 conferred a ten-fold increase in the risk of 1-year mortality (HR 10.24; 95% CI 7.94–11.45; *p* < 0.0001), and hypoalbuminemia was associated with a four-fold increase (HR 4.23; 95% CI 2.59–4.16; *p* = 0.0043). Delayed surgery and elevated NT-proBNP levels were similarly confirmed to be independent risk factors for adverse outcomes.

## 4. Discussion

### 4.1. Background

Fractures of the hip among the elderly are characterized by severe morbidity, longer hospital stays, and elevated mortality rates and are, thus, of paramount interest for optimizing management strategies and outcomes [1,7,18]. Classical factors predicting a poorer prognosis following hip fracture have included advanced age, comorbidity quantified by the Charlson Comorbidity Index (CCI), hypoalbuminemia, and markers of cardiac impairment such as NT-proBNP [7]. These factors have been thoroughly tested by the literature [16]. In the past few years, the role of background inflammatory response as a predictive factor gained growing attention [19]. Of the reported biomarkers, the NLR and PLR have appeared of interest as markers due to the fact that they reflect both innate immunity activation and adaptive immunity depression, mechanisms implied to be implicated in frailty, healing failure, and postoperative complications [20]. Studies have consistently shown that increased NLR and PLR values are correlated with poorer outcomes across different clinical conditions such as those undergoing cancer surgery, those suffering from cardiovascular disease and sepsis [9,10,11,12,13], etc. Their utility as predictors in the specific context of hip fracture surgery among elderly patients has, however, remained inadequately investigated [14]. In particular, few studies have examined the independent contribution of these markers to short- and long-term mortality and the necessity for intensive care unit admission and hospital length of stay, the most relevant endpoints for both clinicians and the health system [18,21,22]. Given the rising prevalence of hip fractures in aging populations worldwide and the escalating healthcare burden associated with their management, there is a growing need for rapid and reliable tools to identify high-risk patients at the time of hospital admission [3,4]. The demographic shift toward older age groups will inevitably result in a higher volume of fragility fractures, placing additional strain on hospitals, rehabilitation services, and social care systems. Therefore, having early and efficient prognostic indicators is not only clinically relevant but also economically essential for guiding the appropriate allocation of limited resources [23]. In this regard, the NLR and PLR offer distinct advantages. Their values can be calculated easily from standard complete blood count tests, which are already part of routine clinical workups in emergency and preoperative settings [8]. This means no additional procedures or costs are required to obtain them, making them highly accessible for use in virtually any hospital environment. In contrast to more expensive or specialized markers, such as NT-proBNP or advanced imaging modalities, the NLR and PLR can be used universally—even in lower-resourced healthcare systems—making them especially valuable as scalable tools for risk stratification [24]. Their availability, affordability, and fast turnaround time from routine laboratory tests confirm the predictive value of the NLR and PLR as a means by which clinicians could have simple tools for optimizing preoperative risk assessment and the individualization of the postoperative management. Moreover, the use of these markers could enhance personalized medicine approaches by enabling tailored surveillance strategies, the early identification of patients who may benefit from ICU-level care, and proactive complication prevention. This is particularly crucial in older adults, whose surgical recovery is often complicated by underlying immunosenescence, malnutrition, and polypharmacy [25].

Ultimately, integrating the NLR and PLR into standard risk models may contribute to more efficient healthcare delivery, reducing avoidable morbidity, hospital readmissions, and overall costs. Further research is warranted to refine cut-off thresholds and validate their use prospectively, but the current body of evidence provides a strong rationale for their inclusion in future perioperative protocols for geriatric hip fracture patients.

### 4.2. Our Findings

This retrospective cohort study evaluated the prognostic value of the admission of the NLR and PLR in elderly patients undergoing hip fracture surgery, with particular attention paid to 30-day, 90-day, and 1-year mortality; need for ICU admission; and prolonged hospital stay. While confirming the established relevance of factors such as age, comorbidity burden, surgical delay, hypoalbuminemia, and elevated NT-proBNP levels, our analysis highlighted the independent and strong association of elevated NLR and PLR values with adverse clinical outcomes. Univariable analysis showed that an NLR greater than 7.2 was significantly associated with increased risk of 30-day mortality (HR 2.38), 90-day mortality (HR 1.92), ICU admission (HR 2.52), and prolonged hospitalization (HR 1.97). Similarly, a PLR above 189.4 predicted poor outcomes, albeit with slightly lower hazard ratios. Multivariable Cox regression models, adjusted for key confounders, confirmed the independent prognostic significance of both markers for 1-year and overall mortality. Our findings suggest that elevated NLR and PLR are not merely byproducts of pre-existing disease but also reflect active pathophysiological processes that adversely influence postoperative recovery and survival. A heightened neutrophilic response may drive catabolism, endothelial dysfunction, and impaired healing, while lymphopenia may signal immunosuppression and increased susceptibility to postoperative complications [12,20]. Likewise, an elevated PLR may indicate a prothrombotic and immunologically imbalanced state known to compromise outcomes after major orthopedic interventions [13,14]. Importantly, the prognostic impacts of elevated NLR and PLR values were comparable to those of established markers such as hypoalbuminemia and NT-proBNP, reinforcing the clinical significance of systemic inflammation in this setting. Given the rapid, cost-effective availability of the NLR and PLR at admission, incorporating these indices into existing risk models could enhance the early identification of high-risk patients and inform targeted perioperative management strategies. Optimal cut-off values for the NLR and PLR were determined through univariable and ROC curve analyses, yielding thresholds of 7.2 and 189.4, respectively. The AUC for the NLR was 0.78 (95% CI 0.66–0.81), demonstrating good discriminatory power, with a sensitivity of 69.7% and a specificity of 85.4%. The AUC for the PLR was 0.73 (95% CI 0.61–0.79), indicating moderate discriminatory ability, with a sensitivity of 65.1% and a specificity of 76.1%. While these results underscore the strong predictive performance of the NLR, and to a lesser extent the PLR, further validation in larger, prospective cohorts is necessary to confirm these cut-offs and extend their applicability across broader clinical contexts.

### 4.3. Clinical Implications

Our findings suggest that the NLR and PLR hold promise as readily available biomarkers for risk stratification in elderly hip fracture patients. Elevated preoperative NLR and PLR values may identify individuals at higher risk of postoperative complications, potentially warranting more intensive preoperative optimization, closer postoperative monitoring, and a tailored approach to rehabilitation. Integrating these ratios into routine clinical assessment could facilitate personalized perioperative management strategies aimed at improving patient outcomes and mitigating the burden of postoperative morbidity in this vulnerable population. Future prospective studies are needed to validate these implications and establish specific clinical protocols based on NLR and PLR values.

### 4.4. Limitations

Several limitations should be acknowledged when interpreting the results of our study. First, its retrospective, single-center design inherently introduces potential biases related to data completeness, selection, and unmeasured confounders. Although multiple imputation techniques and rigorous multivariable adjustments were employed, residual confounding cannot be entirely excluded. Second, while the NLR and PLR were measured at the time of emergency department admission, dynamic changes in these markers over the perioperative period were not assessed. It is possible that serial measurements might provide even greater prognostic information compared to single time-point assessments. Third, differences in laboratory techniques, patient characteristics, and healthcare delivery systems could affect the reproducibility of our findings. Lastly, cause-specific mortality was not distinguished from all-cause mortality in our study, which might limit the ability to attribute death directly to surgical complications or systemic decompensation. Despite these limitations, the consistency, strength, and clinical plausibility of the associations observed support the robustness of our conclusions and provide a compelling rationale for future prospective studies focusing on the role of systemic inflammation in postoperative outcomes among elderly hip fracture patients.

## 5. Conclusions

Our study shows that high admission levels of the NLR and PLR are strong, independent predictors of short- and long-term unfavorable outcomes in elderly patients undergoing hip fracture surgery. Whereas conventional risk factors like chronological age, comorbid burden, nutritional status, and cardiac function are still indispensable in risk appraisal, supplementary simple and widely available inflammatory markers like the NLR and PLR have the potential to significantly contribute to prognostic stratification. These hematologic indices are easily derived from routine blood work, do not require additional testing or cost, and can be obtained within hours of hospital admission. Their accessibility makes them especially attractive for real-world clinical settings, including those with limited resources. The NLR and PLR not only reflect the inflammatory and immune status of the patient but also correlate with physiological stress responses, systemic inflammation, and impaired recovery potential, all of which are relevant in the perioperative period. Incorporating the NLR and PLR into existing risk models could provide a more nuanced understanding of patient vulnerability. This is especially pertinent as the population continues to age and the incidence of hip fractures increases globally, placing additional strain on healthcare systems. The efficient and early identification of high-risk individuals enables personalized interventions, such as enhanced monitoring, pre-emptive medical optimization, or prioritization for early surgery. The NLR and PLR may potentially be applied to routine clinical practice to inform perioperative care, streamline resource utilization, and ultimately better care for this frail group of patients. By improving early risk stratification with these inexpensive tools, clinicians may also reduce the likelihood of complications, shorten hospital stays, and improve both clinical and economic outcomes in elderly patients recovering from hip fractures.

## Figures and Tables

**Table 1:** The baseline characteristics of the study population.

Characteristics	Total (N = 395)
Age, years, mean (SD)	84 (12.3)
Female, n (%)	223 (56.4)
CCI score, n (%)	
CCI = 0	144 (36.8)
CCI = 1	137 (34.2)
CCI > 2	114 (29.0)
Type of fracture, n (%)	
Subcapital	112 (28.5)
Basi-cervical	98 (24.8)
Intertrochanteric	185 (46.7)
Type of surgery, n (%)	
Hemiarthroplasty	73 (18.3)
Total hip arthroplasty	54 (13.6)
Cannulated screw fixation	15 (3.8)
Internal fixation	253 (64.3)
Time to surgery, days, median (IQR)	2.9 (0.5–5.9)
Surgery within 48 h, n (%)	289 (72.4)
Surgery after 48 h, n (%)	106 (27.6)
Pre-trauma anticoagulant therapy, n (%)	93 (24.2)
NLR, median (IQR)	6.73 (2.4–9.2)
PLR, median (IQR)	122.5 (112.5–197.8)
Hemoglobin, g/dL, median (IQR)	10.2 (6.7–14.9)
NT-proBNP, ng/L, median (IQR)	390.5 (187.3–14,573.2)
Albumin, g/dL, median (IQR)	4.6 (2.5–5.2)
Hypoalbuminemia (<3.5 g/dL), n (%)	82 (21.3)
30-day mortality, n (%)	19 (4.8)
90-day mortality, n (%)	40 (10.5)
1-year mortality, n (%)	53 (13.9)
Total mortality, n (%)	112 (28)
ICU admission after surgery, n (%)	37 (9.6)
Prolonged hospitalization (>15 days), n (%)	61 (15.9)
Length of stay, days, mean (SD)	9 (3.2)

SD, standard deviation; IQR, interquartile range; HR, hazard ratio; CI, confidence interval; CCI, Charlson Comorbidity Index; NLR, neutrophil-to-lymphocyte ratio; PLR, platelet-to-lymphocyte ratio; NT-proBNP, N-terminal pro-B-type natriuretic peptide; ICU, intensive care unit; LoS, length of stay.

**Table 2:** Univariable Cox regression analysis for predictors of 30-day and 90-day mortality, ICU admission, and prolonged hospitalization.

Variables	30-Day Mortality HR (95% CI)	*p*-Value	90-Day Mortality HR (95% CI)	*p*-Value	ICU Admission HR (95% CI)	*p*-Value	LoS > 15 Days HR (95% CI)	*p*-Value
Age (per 1-year increase)	1.32 (1.02–1.37)	0.0023	1.38 (1.12–1.42)	0.0017	1.03 (0.98–1.12)	0.059	1.89 (1.42–2.02)	0.0019
CCI > 2	8.73 (6.84–9.43)	0.0001	12.73 (9.89–13.32)	0.003	7.29 (6.23–9.21)	0.0012	6.74 (5.34–8.21)	0.0018
Time to surgery (per 1-day increase)	1.12 (1.01–1.43)	0.417	1.35 (1.07–1.52)	0.004	1.12 (0.99–1.24)	0.061	1.59 (1.21–1.79)	0.003
Hypoalbuminemia (<3.5 g/dL)	3.23 (2.89–3.76)	0.0043	4.25 (3.39–4.78)	0.0023	1.43 (1.23–1.67)	0.0012	1.04 (0.98–1.12)	0.074
NT-proBNP > 1099 ng/L	1.37 (1.14–1.78)	0.001	1.45 (1.10–1.79)	0.002	1.17 (1.01–1.49)	0.046	1.29 (1.12–1.56)	0.039
NLR > 7.2	2.38 (2.01–2.75)	0.001	1.92 (1.23–2.13)	0.003	2.52 (2.21–2.87)	0.0023	1.97 (1.23–2.17)	0.002
PLR > 189.4	1.99 (1.73–2.31)	0.002	1.78 (1.43–1.99)	0.002	1.65 (1.23–1.89)	0.0021	1.54 (1.21–1.78)	0.0052

SD, standard deviation; IQR, interquartile range; HR, hazard ratio; CI, confidence interval; CCI, Charlson Comorbidity Index; NLR, neutrophil-to-lymphocyte ratio; PLR, platelet-to-lymphocyte ratio; NT-proBNP, N-terminal pro-B-type natriuretic peptide; ICU, intensive care unit; LoS, length of stay.

**Table 3:** Multivariable Cox regression analysis for 1-year mortality and total mortality.

Variables	1-Year Mortality HR (95% CI)	*p*-Value	Total Mortality HR (95% CI)	*p*-Value
Age (per 1-year increase)	1.13 (1.01–1.39)	0.067	1.10 (0.98–1.52)	0.057
CCI > 2	10.24 (7.94–11.45)	0.0001	11.24 (9.79–13.21)	0.002
Time to surgery (per 1-day increase)	1.27 (1.11–1.53)	0.017	1.37 (1.12–1.62)	0.004
Hypoalbuminemia (<3.5 g/dL)	4.23 (2.59–4.16)	0.0043	4.64 (3.19–4.88)	0.0023
NT-proBNP > 1099 ng/L	1.27 (1.04–1.68)	0.004	1.38 (1.09–1.78)	0.002
NLR > 7.2	1.98 (1.73–2.15)	0.001	1.95 (1.27–2.23)	0.003
PLR > 189.4	1.81 (1.61–2.23)	0.032	1.68 (1.29–1.83)	0.029

SD, standard deviation; IQR, interquartile range; HR, hazard ratio; CI, confidence interval; CCI, Charlson Comorbidity Index; NLR, neutrophil-to-lymphocyte ratio; PLR, platelet-to-lymphocyte ratio; NT-proBNP, N-terminal pro-B-type natriuretic peptide; ICU, intensive care unit; LoS, length of stay.

## Data Availability

The datasets generated and/or analyzed during the current study are not publicly available due to institutional data protection policies but are available from the corresponding author upon reasonable request.

## Abbreviations

The following abbreviations are used in this manuscript:

AUC	Area Under the Curve
CCI	Charlson Comorbidity Index
CI	Confidence Interval
ED	Emergency Department
HR	Hazard Ratio
ICU	Intensive Care Unit
IQR	Interquartile Range
IRB	Institutional Review Board
LoS	Length of Stay
NLR	Neutrophil-to-Lymphocyte Ratio
NT-proBNP	N-terminal pro–B-type Natriuretic Peptide
PLR	Platelet-to-Lymphocyte Ratio
ROC	Receiver Operating Characteristic
SD	Standard Deviation
STROBE	Strengthening the Reporting of Observational Studies in Epidemiology
